# Long-chain acyl-CoA synthetase 2 is involved in seed oil production in *Brassica napus*

**DOI:** 10.1186/s12870-020-2240-x

**Published:** 2020-01-13

**Authors:** Li-Na Ding, Shou-Lai Gu, Fu-Ge Zhu, Zhong-Yan Ma, Juan Li, Ming Li, Zheng Wang, Xiao-Li Tan

**Affiliations:** Institute of Life Sciences, Jiangsu University, Zhenjiang, China

**Keywords:** *BnLACS2*, Oil contents, Glycolysis, Fatty acids, Lipid, Rapeseed (*Brassica napus*)

## Abstract

**Background:**

Triacylglycerols (TAGs) are the main composition of plant seed oil. Long-chain acyl-coenzyme A synthetases (LACSs) catalyze the synthesis of long-chain acyl-coenzyme A, which is one of the primary substrates for TAG synthesis. In *Arabidopsis*, the LACS gene family contains nine members, among which *LACS1* and *LACS9* have overlapping functions in TAG biosynthesis. However, functional characterization of LACS proteins in rapeseed have been rarely reported.

**Results:**

An orthologue of the *Arabidopsis LACS2* gene (*BnLACS2*) that is highly expressed in developing seeds was identified in rapeseed (*Brassica napus*). The *Bn*LACS2-GFP fusion protein was mainly localized to the endoplasmic reticulum, where TAG biosynthesis occurs. Interestingly, overexpression of the *BnLACS2* gene resulted in significantly higher oil contents in transgenic rapeseed plants compared to wild type, while *BnLACS2*-RNAi transgenic rapeseed plants had decreased oil contents. Furthermore, quantitative real-time PCR expression data revealed that the expression of several genes involved in glycolysis, as well as fatty acid (FA) and lipid biosynthesis, was also affected in transgenic plants.

**Conclusions:**

A long chain acyl-CoA synthetase, *Bn*LACS2*,* located in the endoplasmic reticulum was identified in *B. napus*. Overexpression of *BnLACS2* in yeast and rapeseed could increase oil content, while *BnLACS2*-RNAi transgenic rapeseed plants exhibited decreased oil content. Furthermore, *BnLACS2* transcription increased the expression of genes involved in glycolysis, and FA and lipid synthesis in developing seeds. These results suggested that *BnLACS2* is an important factor for seed oil production in *B. napus*.

## Background

Oilseed rape *(Brassica napus.* L) is one of the four major oil plants in the world, and plays an important role in producing vegetable proteins and edible oils for human consumption. Moreover, seed oils are important raw materials for biofuels and in the pharmaceutical industry with substantial economic value and high demand in recent years [1, 2]. Thus, increasing the oil content of seeds is important for genetic breeders and has become a major topic of oil crop research [3, 4].

Vegetable oil is mainly accumulated during the seed maturation phase and supplies carbon and energy for seed germination and seedling growth [5, 6]. Seed oils mainly consist of triacylglycerols (TAGs), which comprise as much as 60% of the weight of a seed, and represent the most efficient form of energy storage in eukaryotic cells [7, 8]. Given the important role for TAGs, understanding the factors that limit their accumulation will contribute to increase TAG content by genetic engineering. In developing oilseeds, TAG biosynthesis takes place in the endoplasmic reticulum (ER). Carbohydrate and fatty acid (FA) metabolism are also involved in this pathway. In the cytosol and plastid, hexose is converted into acetyl-CoA through the glycolytic pathway, of which some are then used as the carbon source for FA synthesis. FAs can be converted to long-chain acyl-CoAs, which are precursors for TAG biosynthesis. Many genes that encode enzymes involved in glycolysis, as well as in FA and lipid biosynthesis play crucial roles in this pathway. Among them, the genes encoding long-chain acyl-CoA synthetase (LACS, EC 6.2.1.3) catalyze the formation of acyl-CoA thioesters from free FAs in the presence of CoA, ATP, and Mg^2+^. It is a critical process in FA metabolism in prokaryotes and eukaryotes, and involves a two-step reaction. In the first step, free FAs react with ATP to generate a acyl-AMP, after which the acyl-CoA thioester bond is formed and AMP is released in the following step [9, 10]. LACSs belong to the AMP binding protein (AMPBP) super family and mainly catalyze the synthesis of acyl-CoAs with acyl chain lengths of 12–20 carbons [11]. Previous studies have demonstrated that LACSs take an important position in nearly all FA-derived pathways, including lipid metabolism, jasmonate biosynthesis and β-oxidation [12, 13], as well as intracellular FA homeostasis and FA transport in various microorganism and mammalian cells [14–16]. In the diatom *Phaeodactylum tricornutum*, a lower photosynhetic organism, there were five putative LACSs (*PtACSL1–5*), and only two of which were able to restore growth, facilitate exogenous FA uptake, and enhance lipid accumulation in the yeast double mutant, *FAA1ΔFAA4Δ* [17]. Thus, those studies have provided a molecular basis for the study of LACS-mediated FA and lipid metabolism in different organisms.

In higher plants, LACSs have several isoforms, which perform diverse biological functions in different cellular compartments [10, 18–21]. In *Arabidopsis*, there are nine *LACS* genes that participate in FA and glycerolipid metabolism. Seven of which could effectively complement the growth phenotype of a LACS-deficient yeast mutant strain, YB525. The two that could not complement the growth phenotype were peroxisomal isoforms [13]. *AtLACS1* and *AtLACS2* localize to the ER and have overlapping functions in wax and cutin synthesis with very long chain acyl-coenzyme A synthetase activity [22–24]. *AtLACS1* and *AtLACS4* showed a synergistic effect in the process of proper pollen coat formation [25]. *AtLACS6* and *AtLACS7* were involved in peroxisomal β-oxidation and successful seedling establishment [10, 26]. *LACS9* is localized to the chloroplast envelope and is involved in the production of acyl-CoA [21], and its function partially overlaps that of *LACS1* and *LACS4* in TAG biosynthesis and lipid trafficking from the ER to the plastid, respectively, in *Arabidopsis* [18, 27]. LACS homologs were also found in other plants, such as rapeseed, rice, soybean, and cotton, among others. A peroxisomal *GmACSL2* from *Glycine max* is probably involved in FA and lipid degradation during seed germination [28]. Sunflower *HaLACS1* and *HaLACS2*, which display sequence homology with the *Arabidopsis LACS9* and *LACS8* genes, respectively, were expressed at high levels in developing seeds and played an important role in sunflower oil synthesis [29].

Previous studies showed that there are six homologous *LACS* genes in *B. napus*, and two of which, including *BnLACS2,* have enzymatic activity when expressed in *E. coli* [30, 31]. However, the biochemical properties and physiological functions of *BnLACS2* remain uncharacterized. In this study, the *B. napus BnLACS2* gene was cloned and its function in seed oil production was investigated. Overexpression of *BnLACS2* in rapeseed plants resulted in improved oil content, while the suppression of gene expression in rapeseed decreased oil content. It was also shown that alterations in *BnLACS2* function was associated with the physiological process of glycolysis, and FA and lipid synthesis in transgenic plants.

## Results

### *BnLACS2* is the orthologue of *Arabidopsis LACS2* in rapeseed

**Fig. 1:**
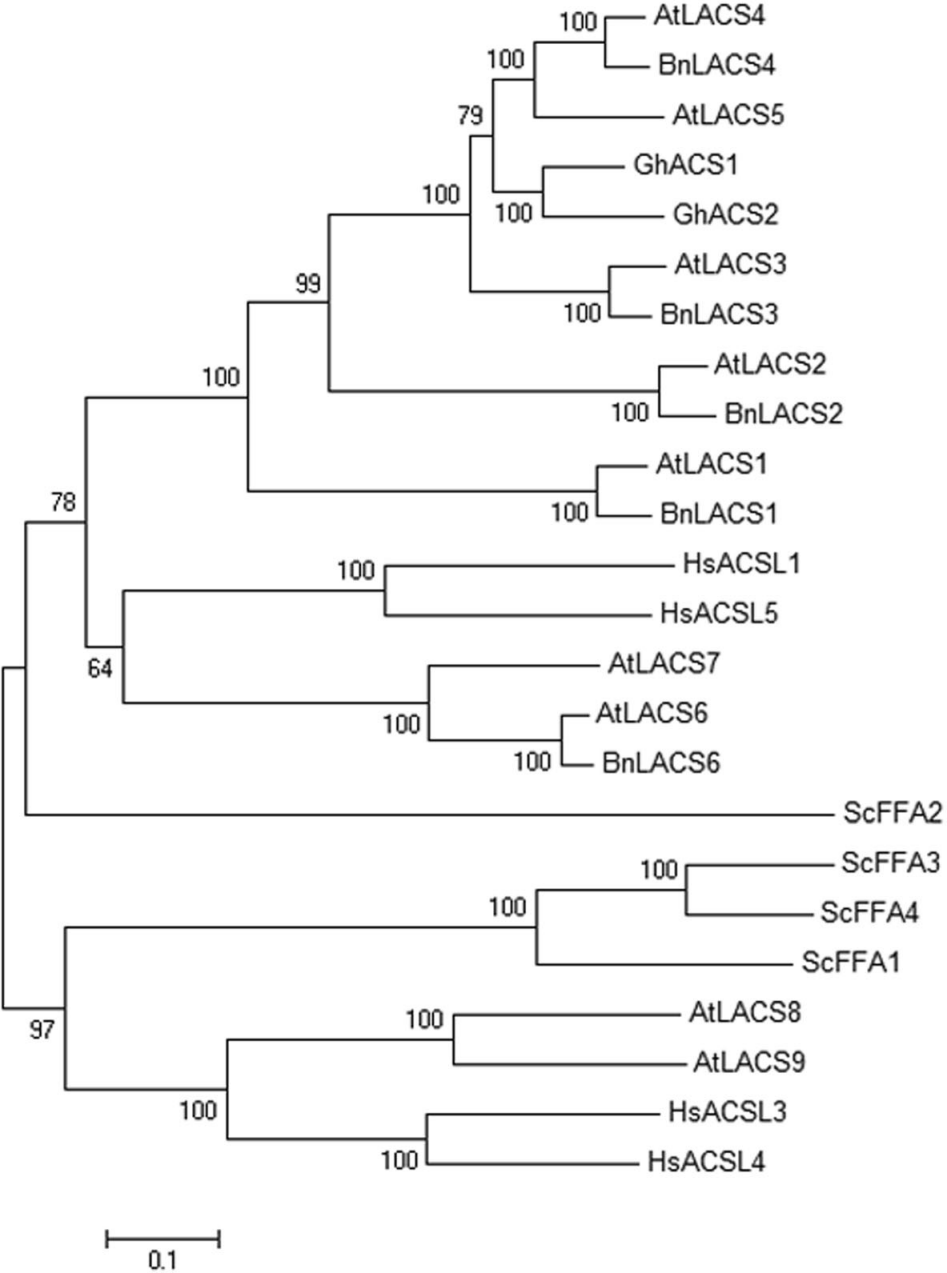
Phylogenetic analysis of LACS protein family. The numbers beside the branches represent bootstrap on 1000 replications. The accession numbers are as follows: *Arabidopsis thaliana At*LACS1 (NP_182246), *At*LACS2 (NP_175368), *At*LACS3 (NP_176622), *At*LACS4 (NP_194116), *At*LACS5 (AAM28872), *At*LACS6 (NP_566265), *At*LACS7 (NP_198112), *At*LACS8 (NP_178516), *At*LACS9 (NP_177882), *Gossypium hisutum Gh*ACS1 (ABA00144), *Gh*ACS2 (ABA00145), *B. napus Bn*LACS1 (BnaC04g51420D), *Bn*LACS2 (BnaA05g16170D), *Bn*LACS3 (BnaC09g11030D), *Bn*LACS4 (BnaC01g15670D), *Bn*LACS6 (BnaA03g29320D), *Homo sapiens Hs*ACSL1 (NP_001986), *Hs*ACSL3 (NP_976251), *Hs*ACSL4 (NP_004449), *Hs*ACSL5 (NP_976313), *Saccharomyces cerevisiae Sc*FFA1 (NP_014962), *Sc*FFA2 (NP_010931), *Sc*FFA3 (NP_012257), *Sc*FFA4 (NP_013974). Bootstrapping was performed to obtain support values for each branch

Using the protein sequence of *At*LACS2 (NP_175368) as a query probe, two highly homologous sequences (*BnaC05g51350D* and *BnaA05g16170D*) were obtained from the *Brassica* genome database, and the putative full-length cDNA (2001 bp) of the *BnaA05g16170D* locus (designated as *BnLACS2*) was cloned from Zhongshuang 9 variety (Additional file 1: Figure S1). *BnLACS2* contained an open reading frame consisting of 667 amino acid residues with a theoretical isoelectric point of 6.07 and a calculated molecular weight of 74.35 kDa. Sequence analysis revealed that *Bn*LACS2 had a conserved AMP-binding domain, which was the characteristic domain of the AMPBP superfamily. Motif analysis of other LACS sequences using ScanProsite Results Viewer software also identified the notable AMP-binding domain, implying that this motif is functionally important for LACSs. The amino acid sequence of the *Bn*LACS2 protein was then aligned with 24 LACS sequences from different species, including *B. napus*, *Gossypium hirsutum*, *Arabidopsis thaliana*, *Saccharomyces cerevisiae,* and *Homo sapiens*. A phylogenetic tree was constructed based on the amino acid sequences and revealed a close genetic relationship between *BnLACS2* and *Arabidopsis LACS2*, suggesting that they might have similar functions (Fig. 1).

### *Bn*LACS2 can complement the YB525 yeast mutant

**Fig. 2:**
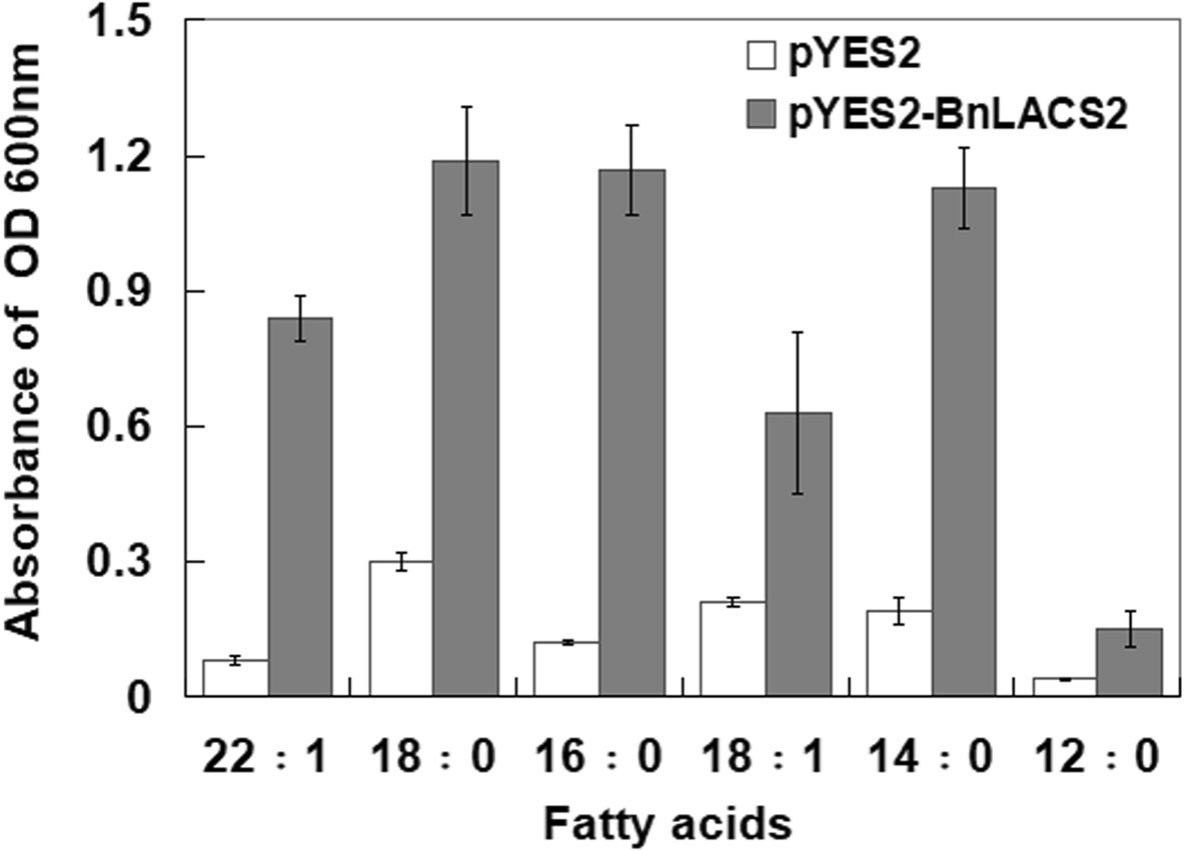
The growth conditions of YB525 cells containing pYES2-*BnLACS2* and pYES2 empty vector. The growth were measured for three lines of pYES2 and pYES2-*BnLACS2* transformants cultured in the liquid medium used the fatty acid 12:0, 14:0, 16:0, 18:0, 18:1 and 22:1 as the sole carbon source, respectively. Error bars indicate SD (*n* = 3)

Previous research showed that *Bn*LACS2 had in vitro enzymatic activity [31]; however, its exact function and mechanism of action have yet to be discovered. The yeast expression system is a powerful tool for the production of recombinant eukaryotic proteins. Moreover, yeast cells can accumulate oil, which is similar to rapeseed oil bodies [32]. Thus, the yeast expression system is suitable for investigating the function of genes in the lipid metabolism pathway. The YB525 yeast mutant lacks acyl-CoA synthetase activity, and provides a unique tool for the assessment of the functional roles of such enzymes [33]. *Arabidopsis* LACS2 exhibited enzymatic activity and could complement the LACS-deficient YB525 strain [13]. To determine whether *Bn*LACS2 had a similar activity in vivo, *BnLACS2* was cloned into pYES2 and transformed into YB525. The transformants were cultured in liquid medium with various FAs as the sole carbon source, including 12:0 lauric acid, 14:0 myristic acid, 16:0 palmitic acid, 18:0 stearic acid, 18:1 oleic acid, and 22:1 erucic acid. Then, the growth of those strains was evaluated based on optical density (OD). As shown in Fig. 2, transformation of pYES2-*BnLACS2* rescued the growth defect of YB525 in medium containing 14:0, 16:0, 18:0, 18:1, and 22:1 FAs, but not 12:0 FAs, which is a short chain FA. However, yeast cells transformed with the pYES2 control vector failed to grow in cultures with FAs as the sole carbon source. Moreover, yeast cells containing the pYES2-*BnLACS2* construct preferred to use 18:0 FAs as a carbon source for growth. Thus, *Bn*LACS2 exhibited acyl-CoA synthetase activity and had a substrate preference for long chain FAs.

### Expression analysis of *BnLACS2* in different tissues and different developmental stages of rapeseeds

**Fig. 3:**
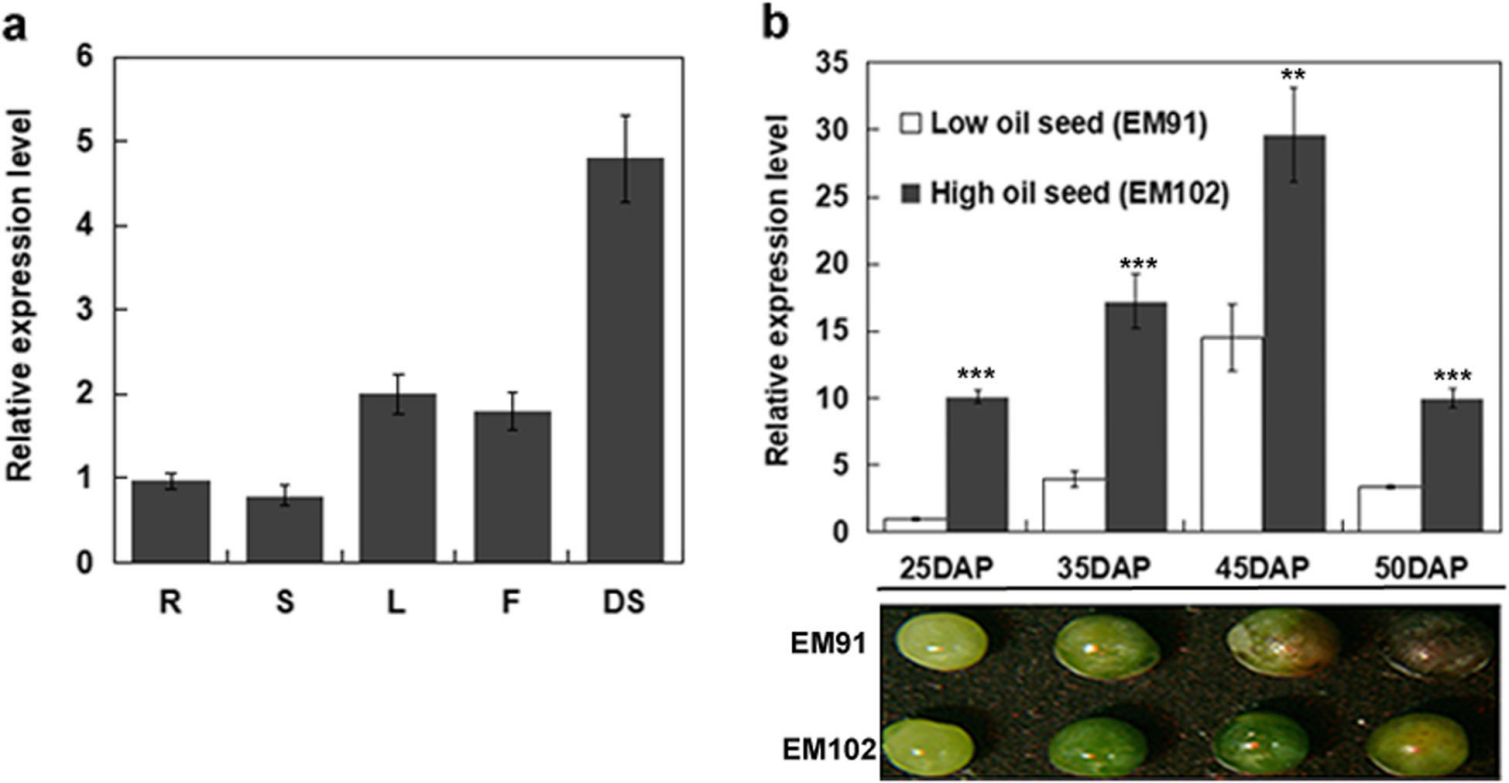
Expression patterns of *BnLACS2*. **a** qRT-PCR analysis of *BnLACS2* in different tissues, including root (R), stem (St), leaf (L), flower (F), developing seed (DS, DAP25). The expression levels were quantified relative to the value obtained from root. **b** Expression patterns of *BnLACS2* in low (EM91) and high (EM102) oil-content seeds of different developmental stages. The photographs were taken during the developing of seeds at 25 DAP, 35 DAP, 45 DAP, and 50 DAP. The expression levels were quantified relative to the value obtained from EM91 (25 DAP). Error bars represent SD (*n* = 3). The significant differences between EM91 and EM102 at each time point (25 DAP, 35 DAP, 45 DAP, and 50 DAP) are indicated (Student’s *t*-test) as follows: ***, *P* < 0.001; **, *P* < 0.01; *, *P* < 0.05

Although *BnLACS2* could activate the free FA, its physiological function is unclear in rapeseed. Considering that the tissue distribution of LACS genes may be closely related to their function, quantitative reverse transcription-polymerase chain reaction (qRT-PCR) was used to detect the expression patterns of the *BnLACS2* gene in different rapeseed tissues, including roots, stems, leaves, flowers and developing seeds 25 days after pollination (DAP). The results showed that the *BnLACS2* gene was expressed in all examined tissues, but was predominantly expressed in developing seeds where TAG was actively synthesized (Fig. 3a). Furthermore, there was an obvious difference in the abundance of *BnLACS2* transcripts between high and low oil-content seeds at 25 DAP, 35 DAP, 45 DAP, and 50 DAP. Regardless of whether expression was evaluated in high or low oil-content seeds, *BnLACS2* was strongly expressed at 45 DAP, which is the stage at which TAG is accumulated at a high rate (Fig. 3b). According to the transcription analysis results described above, *BnLACS2* might be involved in supplying acyl-CoAs for TAG biosynthesis during seed development.

### Subcellular localization of *Bn*LACS2

**Fig. 4:**
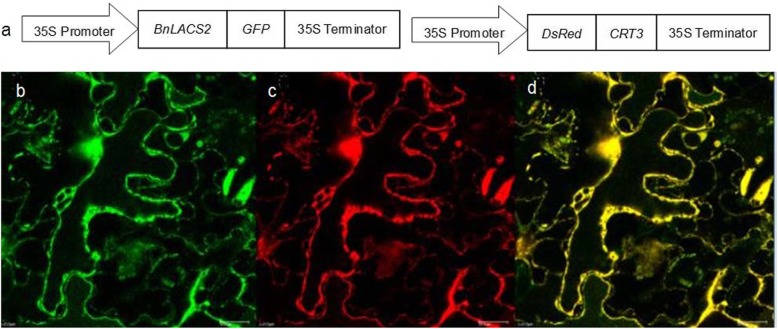
Subcellular localization of *Bn*LACS2. **a** Schematic representation of the *35S:BnLACS2-GFP* and *35S:DsRed-CRT3* construct. **b**-**d** Confocal micrograph of leaf epidermal cells of *N. benthamiana* transiently expressing the *35S:BnLACS2-GFP* (**b**) and *35S:DsRed-CRT3* (**c**) constructs. The merged image of a and b is shown in (**d**). Bar = 20 μm

In higher plants, LACS proteins with different subcellular localizations may participate in different metabolic pathways. It is known that the ER is an important site for FA carbon chain elongation and lipid synthesis. *At*LACS2 is homologous to *Bn*LACS2 and located in the ER. Therefore, we speculated that *Bn*LACS2 is also an ER protein. To determine its localization, calreticulin 3 (CRT3) was selected as a marker for ER localization [34] and fused with a red fluorescent protein (DsRed) (Fig. 4a right). *Bn*LACS2 was expressed as a fusion protein with green fluorescent protein (GFP) using the pK7FWG2.0 vector under the control of the CaMV 35S promoter (Fig. 4a left). The plasmids, *35S::BnLACS2-GFP* and *35S::DsRed-CRT3*, were generated and transfected into the leaf epidermal cells of *Nicotiana benthamiana* for transient expression. As shown in Fig. 4b-d, the GFP fluorescence produced by the *Bn*LACS2-GFP fusion protein was found to overlap with the DsRed fluorescence produced by DsRed-CRT3 fusion protein, indicating that *Bn*LACS2 was co-localized with CRT3 in the ER, i.e., the site of TAG biosynthesis.

### *BnLACS2* expression is correlated with lipid and FA contents in transgenic yeast and rapeseed

Expression pattern and subcellular localization analyses of *Bn*LACS2 suggested that it may be involved in seed oil production. The function of *Bn*LACS2 was first identified with *S. cerevisiae pep4* mutants with at least 90% reduced activity of the four vacuolar proteinases [35]. The Sudan black B staining method was used to indicate the relative content of neutral lipids in host yeast cells. The results showed that the absorbance values of *BnLACS2*-transformed lines increased by 85.7%, compared to the control cell transformed with empty pYES2. Moreover, *BnLACS2* overexpression resulted in producing new phospholipid components in yeast. According to gas chromatography-mass spectrometry (GC-MS) analyses, heterologous expression of *BnLACS2* also significantly enhanced the synthesis of total FAs, and in particular, two main FA components, palmitoleic acid (C16:1) and oleic acid (C18:1) (Additional file 2: Figure S2).

**Fig. 5:**
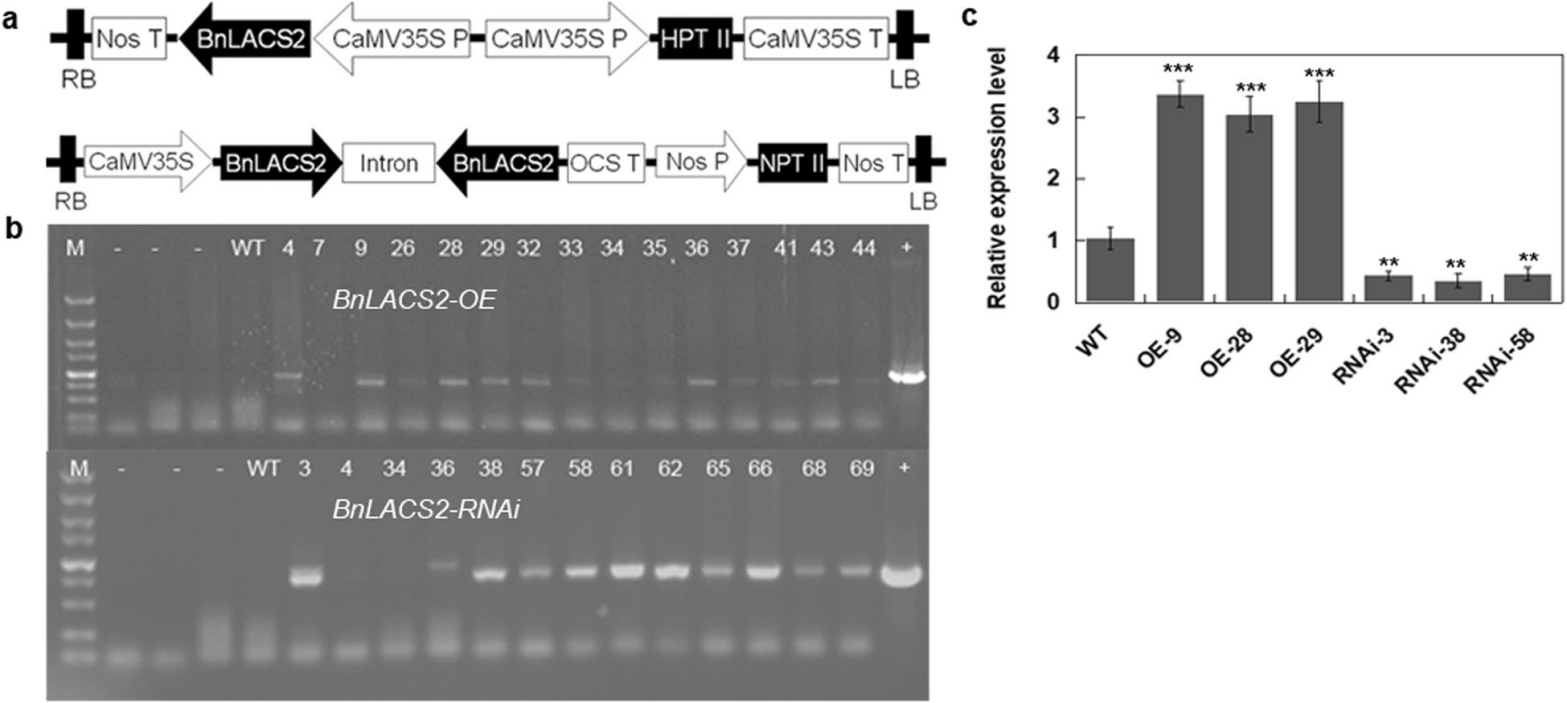
Identification of transgenic rapeseed lines. **a** Schematic diagram (not to scale) of a part the T-DNA region of *BnLACS2*-OE and *BnLACS2*-RNAi constructs used to transform canola. **b** PCR detection of positive *BnLACS2*-OE and *BnLACS2*-RNAi transgenic plants from a small number of resistant plants. **c** qRT-PCR analysis of *BnLACS2* in different transgenic plant lines. The expression levels were quantified relative to the value obtained from WT plants. Error bars represent SD (*n* = 3). WT, non-transgenic canola; +, positive plasmid DNA; −, negative ddH_2_O, Lane 4–44, *BnLACS2*-OE transgenic plants, Lane 3–69, *BnLACS2*-RNAi transgenic plants

**Fig. 6:**
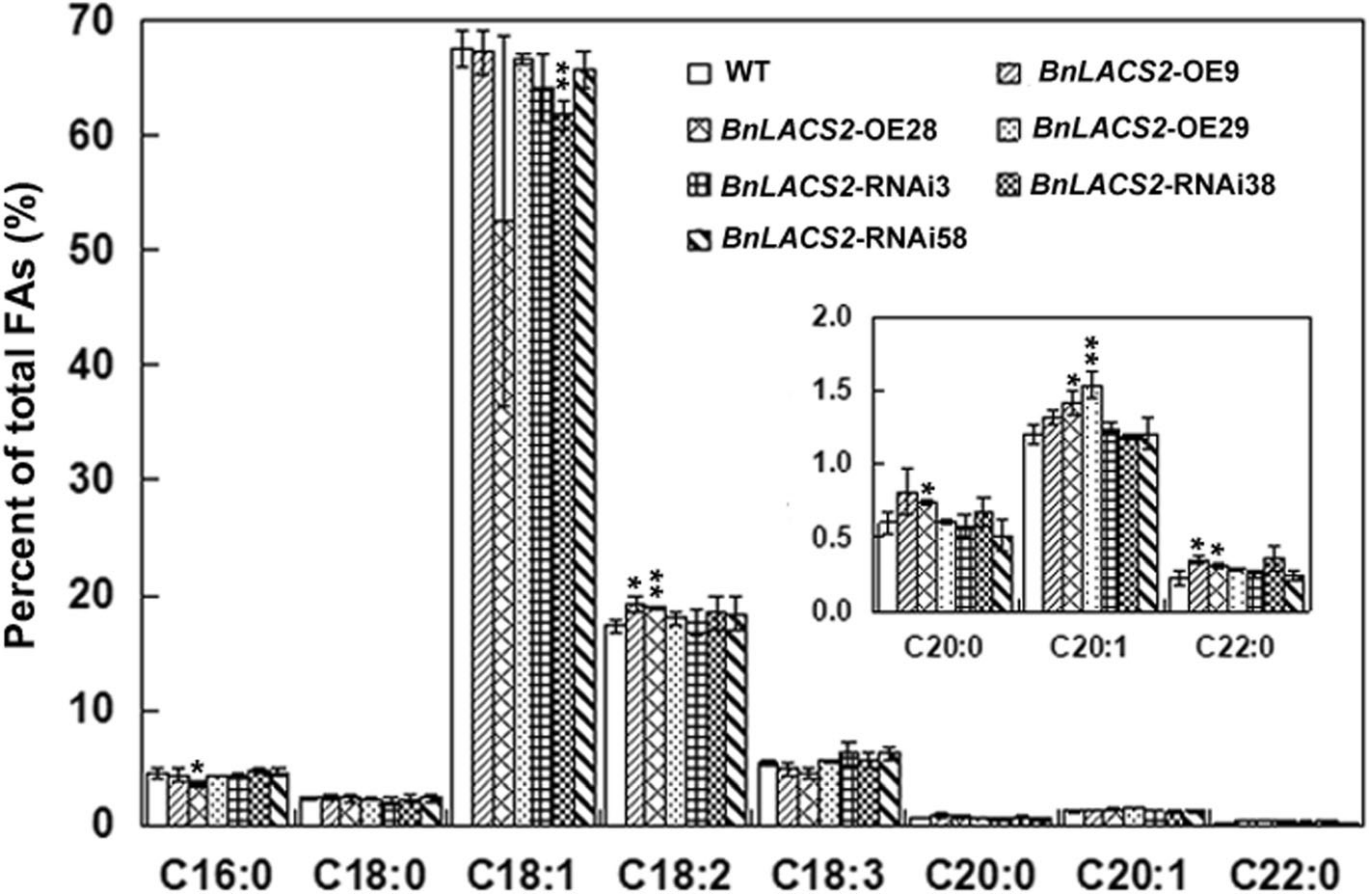
Analysis of FA contents in WT, *BnLACS2*-OE and *BnLACS2*-RNAi transgenic seeds. Mature T_2_ generation seeds were used for the analysis. Error bars represent SD (*n* = 3). The significant differences between transgenic lines and WT are indicated (Student’s *t*-test): **, *P* < 0.01; *, *P* < 0.05

To further confirm the function of *BnLACS2*, vectors for *BnLACS2*-overexpression (OE) and *BnLACS2*-RNAi were constructed and transformed into rapeseed (*B. napus* L. cv. Zhongshuang 9) using the floral dip method (Fig. 5a). The preliminary screening of hygromycin- or kanamycin-resistant rapeseed transformants was performed via PCR. Several independently transformed T0 transgenic plants were selected and grown to maturity to obtain homozygous lines (Fig. 5b). The detection of the *BnLACS2* transcripts by qRT-PCR in T2 transgenic lines revealed that its expression level was higher in *BnLACS2*-OE transgenic plants than in untransformed control plants, while expression was lower in *BnLACS2*-RNAi transgenic plants, compared to the controls (Fig. 5c). The phenotypes of *BnLACS2*-OE and *BnLACS2*-RNAi transgenic plants were also analyzed. Compared to non-transgenic plants, transgenic plants had normal phenotypes and exhibited no obvious changes in morphology. To further determine whether *BnLACS2* was involved in seed TAG biosynthesis, we analyzed the seed oil content in T2 transgenic rapeseed plants using near infrared spectrometry (NIRS) combined with nuclear magnetic resonance (NMR), and obtained consistent results between these two methods. As shown in Tables 1 and 2, the oil content in *BnLACS2*-OE plants was increased by 6 to 8%, and decreased by 3 to 6% in *BnLACS2*-RNAi transgenic plants, compared to wild type plants. Moreover, GC-MS analysis showed that overexpression of *BnLACS2* also significantly enhanced the contents of long-chain FAs, such as C18:2, C20:0, C20:1 and C22:0 (Fig. 6). These findings suggested that the expression of *BnLACS2* in rapeseed and yeast cells increased the content of lipids and FAs. Thus, as an important enzyme for the catalysis of free FAs to long-chain acyl-CoAs, *BnLACS2* may play an essential role in seed oil production in rapeseed.

**Table 1:** Canola oil content is determined in NMR

Line	Lipid content 1(%)	Lipid content 2(%)	Average of lipid content (%)
WT	38.99	38.94	38.97
*BnLACS2*-OE9	42.28^*^	42.01^*^	42.15^*^
*BnLACS2*-OE28	41.14	41.33	41.37
*BnLACS2*-OE29	42.04^*^	41.98^*^	42.01^*^
*BnLACS2*-RNAi3	36.98^*^	36.84^*^	36.91^*^
*BnLACS2*-RNAi38	37.83	37.77	37.80
*BnLACS2*-RNAi58	36.87^*^	36.60^*^	36.74^*^

The values are the mean percentages of each genotype (*n* = 3). ^*^Statistically significant difference from WT (Students’*t* test, *P* < 0.05)

**Table 2:** Canola oil content is determined by NIRS

Line	Oleic acid	Linoleic acid	Linolenic acid	Erucic acid	SFA	Thioglycoside	Protein	Water	Lipid
WT	59.98	21.47	6.12	0.21	6.59	15.59	25.49	7.34	37.62
*BnLACS*2-OE9	61.10	20.55	7.22	0.11	6.76	16.29	21.98^*^	7.37	39.64^*^
*BnLACS2*-OE28	57.10^*^	19.39^*^	6.32	0.37^*^	6.56	11.68^*^	23.31^*^	6.95	40.05^*^
*BnLACS2*-OE29	60.96	19.81	6.62	0.44^*^	6.71	13.16^*^	21.42^*^	7.13	40.40^*^
*BnLACS2*-RNAi3	56.26^*^	23.32^*^	6.79	0.28	6.78	17.12	24.84	7.69	35.34^*^
*BnLACS2*-RNAi38	56.41^*^	21.54	7.79	0.53^*^	6.45	23.89^*^	26.24	7.35	36.69
*BnLACS2*-RNAi58	57.21^*^	23.16^*^	6.88	0.46^*^	6.67	18.88^*^	24.90	7.85	35.35^*^

The values are the mean counts of triplicate determinations. ^*^Statistically significant difference from WT (Students’*t* test, *P* < 0.05)

### *BnLACS2* overexpression influences the expression of genes involved in glycolysis in developing seeds

**Fig. 7:**
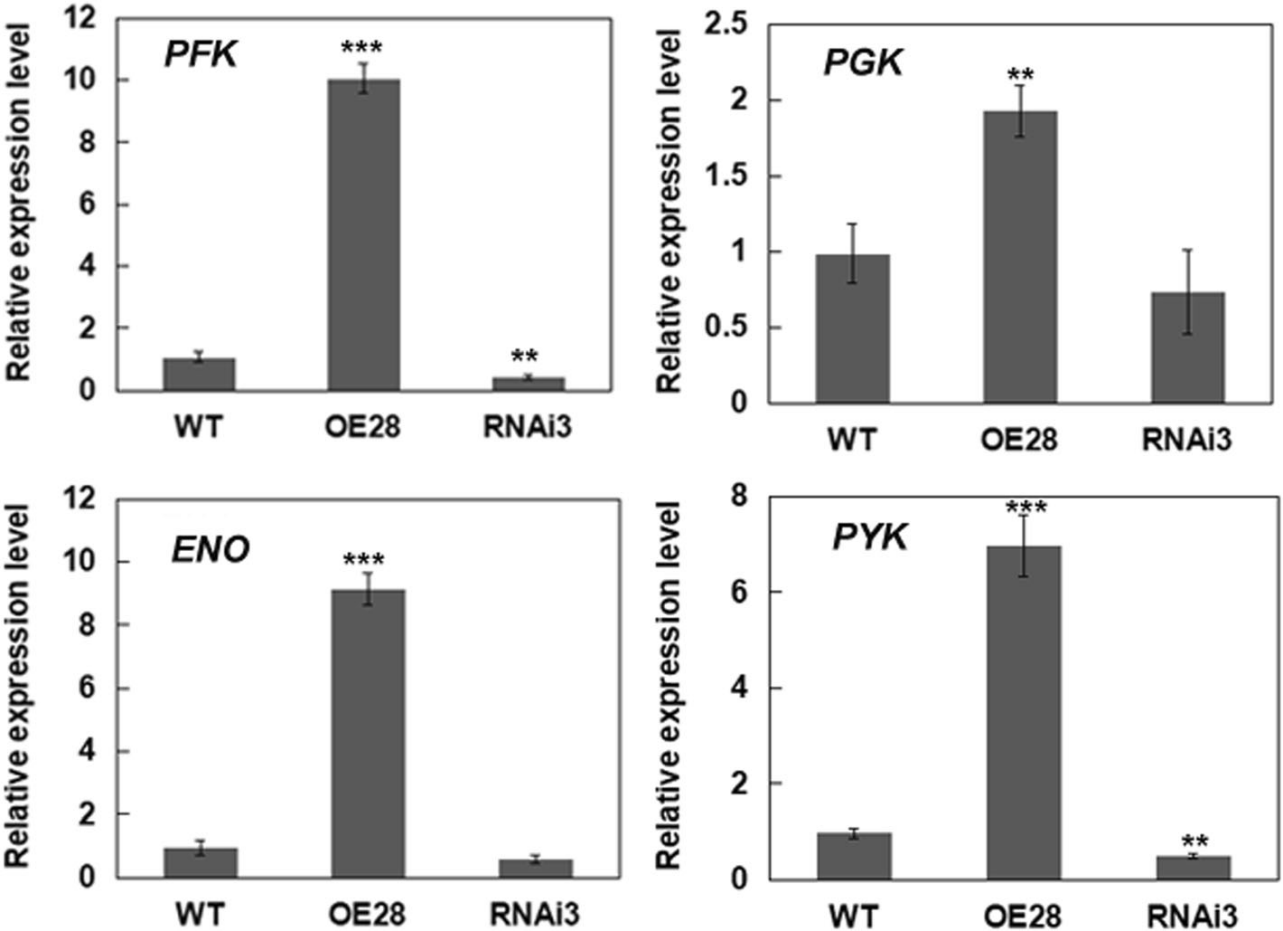
Expression of representative glycolytic genes in developing seeds of the transgenic canola plants. qRT-PCR was performed using total RNA prepared from developing seeds (40 DAP) collected from the WT and transgenic plants. The expression levels were quantified relative to the value obtained from WT plants. Error bars represent SD (*n* = 3). The significant differences between transgenic lines and WT are indicated (Student’s *t*-test): ***, *P* < 0.001; **, *P* < 0.01; *, *P* < 0.05

The expression of *BnLACS2* in yeast *pep4* could increase lipid and FA content. To further characterize the effect of *BnLACS2* in the control of lipid and FA synthesis, the proteins of pYES2 and pYES2-*BnLACS2* transformants were separated using two-dimensional gel electrophoresis (2-DE). The results revealed that some glycolytic enzymes were upregulated in pYES2-*BnLACS2* transformants (Additional files 3 and 4: Figure S3 and Table S1). The upregulation of glycolytic enzymes caused by the expression of *BnLACS2* in yeast prompted us to then detect whether these genes were altered in *BnLACS2* transgenic rapeseed plants. qRT-PCR analysis revealed that the expression of genes for key glycolytic enzymes, including *phosphofructokinase (PFK*), *phosphoglycerate kinase* (*PGK*), *enolase* (*ENO*) and *pyruvate kinase* (*PYK*) [36–38] were significantly increased in *BnLACS2*-OE transgenic plants, while the expression of those genes were decreased in *BnLACS2*-RNAi transgenic plants, compared to non-transgenic plants (Fig. 7). This result indicated that the levels of *Bn*LACS2 affect the expression of glycolytic genes in developing seeds.

### High levels of *Bn*LACS2 activity modifies the expression of FA and lipid synthesis genes in developing seeds

**Fig. 8:**
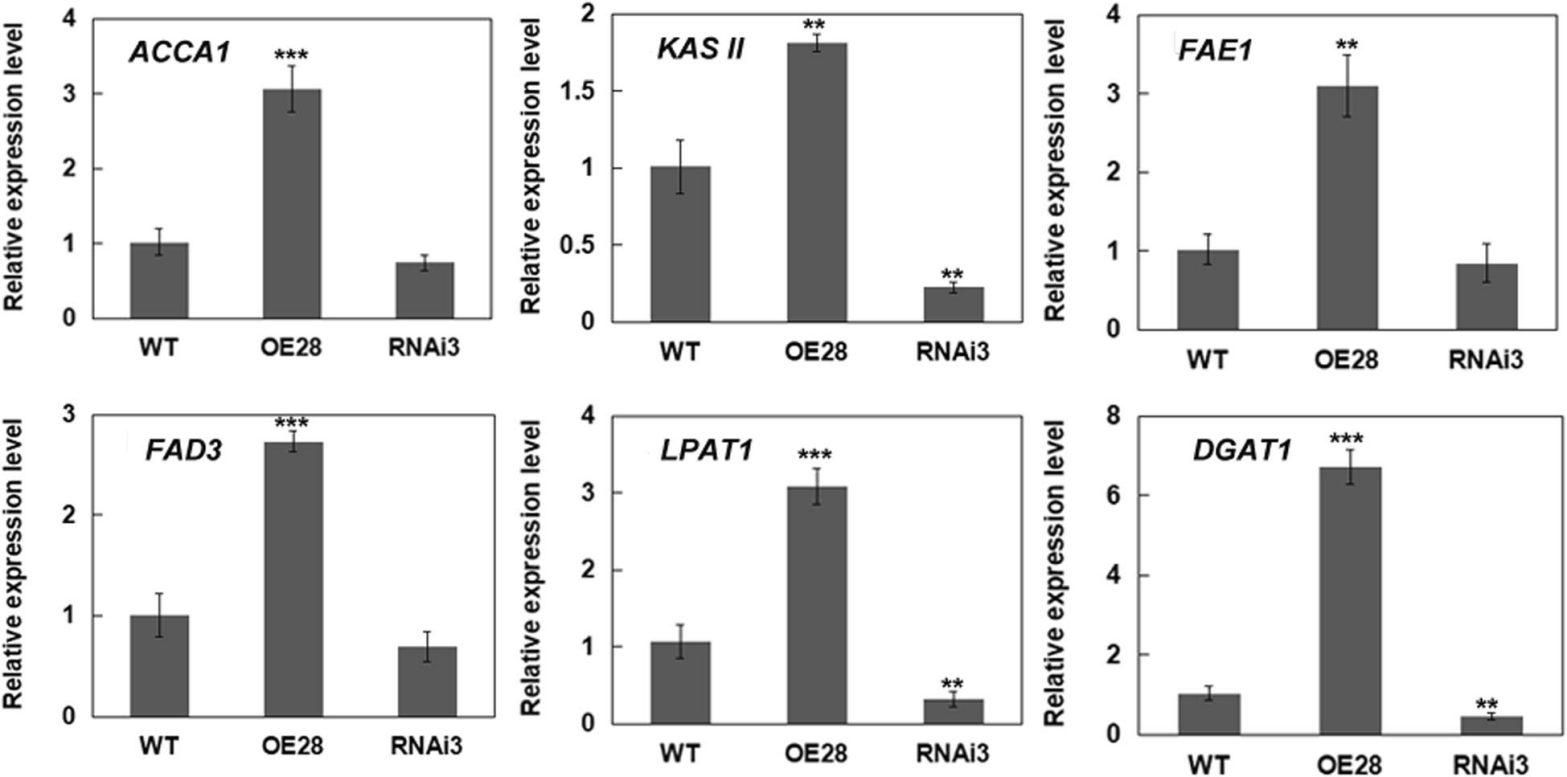
Expression of representative FA and lipid synthetic genes in developing seeds of the transgenic canola plants. The expression levels were quantified relative to the value obtained from WT plants. Error bars represent SD (*n* = 3). The significant differences between transgenic lines and WT are indicated (Student’s *t*-test): ***, *P* < 0.001; **, *P* < 0.01; *, *P* < 0.05

From the above results, we found that modification of *Bn*LACS2 activity levels affected glycolytic gene expression, but the mechanism for that effect is unknown. To further understand the role of *BnLACS2* in the control of seed oil production, the expression level of several representative genes in the FA and lipid biosynthesis pathways were also examined in developing seeds, including *acetyl-CoA carboxylase 1* (*ACC1*), *beta-ketoacyl-[acyl carrier protein]* (ACP) *synthase* (*KAS II*), *FATTY ACID ELONGATION1* (*FAE1*), *FATTY ACID DESATURASE 3* (*FAD3*), *lysophosphatidyl acyltransferase 1* (*LPAT1*), and *diacylglycerol acyltransferase 1* (*DGAT1*). Among those genes, *ACC1* and *KASII* are involved in the FA condensation reaction [39, 40], and *FAE1* and *FAD3* participate in FA chain elongation and desaturation reactions, respectively [41, 42]. *LPAT1* and *DGAT1* are related to lipid biosynthesis [43, 44]. Expression profile analysis showed that those genes were all significantly increased in *BnLACS2*-OE transgenic plants and decreased in *BnLACS2*-RNAi transgenic plants, compared to wild type plants (Fig. 8). These results suggested that high levels of *Bn*LACS2 could modify the expression of FA and lipid synthesis genes in developing seeds and play an important role in seed oil production, although other LACS activities should be involved due to the limited observed changes (±10% of the total oil content).

## Discussion

Oilseed rape is a major oil crop in the world and plays important roles in economy and agricultural production. Increasing seed oil content is of great economic value and a challenge for rapeseed breeding programs. LACSs are important factors for FA and lipid metabolism. Many LACS proteins have been studied in the model plants, *Arabidopsis* and rice [13, 45]. However, functional characterizations of LACS proteins in rapeseed have been rarely reported. In the present study, the LACS gene, *BnLACS2* was identified and characterized from rapeseed (Zhongshuang 9). We showed that *BnLACS2* encodes a LACS protein, which can restore normal growth in a LACS-deficient yeast strain (YB525) upon complementation. Moreover, overexpression of *BnLACS2* in yeast and oilseed rape could significantly increase lipid content. Thus, *BnLACS2* is a candidate gene for high-oil-content breeding in rapeseed.

*Bn*LACS2 contained the conserved AMP-binding domain that is typical of the AMPBP gene superfamily, which share a mechanism step in carboxylic acid activation via adenylation [46]. Phylogenetic analyses showed that *Bn*LACS2 was homologous to *Arabidopsis AtLACS2* (Fig. 1), which has been shown to rescue growth defects of LACS-deficient yeast (YB525) [13]. Based on the sequence homology between *BnLACS2* and *AtLACS2*, it was speculated that they may exhibit similar functions. As a result, it was shown that heterogeneous expression of *BnLACS2* could also complement the YB525 yeast mutant. The yeast complementation test not only confirmed the long-chain acyl-CoA synthetase activity of *Bn*LACS2, but also revealed its substrate preference and specificity. When YB525 yeast cells were transformed with *BnLACS2*, they were able to survive in auxotrophic medium containing FAs of different carbon chain lengths (C12-C22). The data revealed that the growth defect associated with the YB525 was rescued with long chain FAs (C14-C22) as the carbon source when *BnLACS2* was expressed, but not with short chain FAs (C12). These results indicated that *Bn*LACS2 exhibited high acyl-CoA synthetase activity and played an important role in the activation of FAs to acyl-CoA. Furthermore, *Bn*LACS2 preferably utilized long chain FAs as the substrates (Fig. 2), and therefore, *BnLACS2* is a long chain acyl-CoA synthetase gene.

Multiple studies reported that several plant LACSs exhibited different expression patterns in different tissues [13, 20, 28, 29]. In our study, *BnLACS2* was predominantly expressed in developing seeds where TAG was actively synthesized (Fig. 3a). Further analyses of *BnLACS2* expression patterns from 25 DAP to 50 DAP of embryo development showed that *BnLACS2* was strongly expressed at 45 DAP, which is the point at which TAG accumulates at a high rate (Fig. 3b). Those data suggested that *BnLACS2* may be involved in metabolic processes related to seed oil production. It has been reported that LACS proteins in plants may be localized to different organelle fractions to determine the function of LACS proteins [18, 30, 47]. Consistent with the above data, subcellular localization analyses showed that GFP-tagged *BnLACS2* and the DsRed-tagged ER localization marker *CRT3* were co-localized, indicating that *BnLACS2* was localized to the site of TAG biosynthesis (Fig. 4). Similarly, a few plant LACS proteins localized in the ER were reported to play roles in TAG biosynthesis, including *Arabidopsis LACS1* and sunflower *LACS2* [27, 29]. Therefore, *BnLACS2* may participate in the activation of long chain FAs for lipid synthesis during seed development. Moreover, *BnLACS2* was expressed at higher levels in leaves, which was consistent with that of *Arabidopsis LACS2* in cutin biosynthesis [23, 24]. However, the function of *AtLACS2* in seed oil biosynthesis remains unclear.

To assess the contribution of *BnLACS2* in TAG production, *BnLACS2*-OE and *BnLACS2*-RNAi transgenic plants were generated and their seed oil contents were analyzed. The results indicated that transgenic rapeseed plants overexpressing the *BnLACS2* gene exhibited higher oil content in seeds, compared to WT, while RNAi-mediated *BnLACS2* silencing resulted in lower oil contents, compared to WT plants (Tables 1 and 2). Similarly, the expression of *BnLACS2* in *pep4* yeast significantly increased lipid and FA contents (Additional file 2: Figure S2). Thus, these results revealed that *BnLACS2* was involved in TAG biosynthesis. Moreover, both *BnLACS2*-OE and *BnLACS2*-RNAi transgenic plants had normal growth and development. Therefore, *BnLACS2* may be a good candidate gene for increasing the seed oil content of rapeseed, without imparting negative effects.

The 2-DE analysis of yeast proteins showed that heterologous expression of *BnLACS2* resulted in expression profile alterations associated with glycolysis (Additional file 4: Table S1). In transgenic rapeseed plants, the overexpression of *BnLACS2* also affected the expression of glycolytic genes (Fig. 7). Moreover, the increased glycolytic activity was accompanied by increases in lipid content in yeast and *BnLACS2* transgenic plants, which may be due to the increased carbon flux to the FA biosynthesis pathway. In the cytosol and plastid, hexose is converted to acetyl-CoA through the glycolytic pathway, and some of which is then used as the carbon source for FA synthesis. This result implied that *BnLACS2* could also affect FA and lipid biosynthesis. Therefore, it is unsurprising that as a consequence of *BnLACS2* regulation, genes involved in FA and lipid synthesis pathways, including *ACC1*, *KASII*, *FAE1*, *FAD3*, *LPAT1*, and *DGAT1*, were upregulated in *BnLACS2* transgenic plants, and downregulated in *BnLACS2*-silenced plants (Fig. 8).

Previous research demonstrated that the transport of FAs involves two processes, including de novo synthesis of plastidial FAs in the form of acyl-ACP, followed by releasing from ACP by a thioesterase, and subsequent export from plastids in the form of acyl-CoA by LACS action; thus, providing an acyl-donor for TAG assembly in the ER [48–50]. Therefore, LACSs mediate de novo FA synthesis in the plastids and lipid biosynthesis in the ER [29, 48]. In addition to the important role of acyl-CoA esters in TAG synthesis, they also serve as metabolic intermediates in acyl chain elongation reactions, β-oxidation, and other lipid metabolism in plants [45]. It is generally considered that the acyl-CoAs that are synthesized on the outer mitochondrial and peroxisomal membranes are used for oxidation [28, 51], while those synthesized in the ER play synthetic roles [27]. However, the partitioning of acyl-CoAs within cells remains unclear. In this study, *Bn*LACS2 was targeted to the ER, suggesting that it was not required for the transport of FAs through the plastid. Meanwhile, the lipid and FA contents of the *pep4* yeast mutant were improved upon *BnLACS2* overexpression; thus, suggesting that *BnLACS2* had the capacity to supply acyl-CoA substrates for TAG synthesis [52]. To balance acyl-CoA pools in the cytoplasm and ER, it is speculated that some acyl-CoA esters are exported to the ER while releasing the CoA in the cytoplasm. Then, long-chain FAs are reactivated to form acyl-CoA esters by LACS, such as *Bn*LACS2; thus, contributing to TAG accumulation in the ER. However, it is just a possible functioning manner by which the overexpression of *BnLACS2* causes increases in lipid contents in yeast and rapeseed, and additional studies are required to confirm this deduction.

## Conclusions

In the present study, *BnLACS2*, an orthologue of the *Arabidopsis LACS2* gene, was characterized as an important factor determining the seed oil content in the tetraploid, *B. napus*. *BnLACS2* overexpression increased oil content in rapeseed, while its silencing by RNAi decreased oil contents, compared to wild type plants. Furthermore, the altered levels of *Bn*LACS2 were correlated with an enhancement or attenuation of glycolysis, and the FA and lipid synthesis pathways in transgenic plants. These results suggested that the *BnLACS2* gene is a potential target for increasing seed oil production in rapeseed via genetic manipulation efforts. Based on our data, we speculate that *Bn*LACS2 levels modify three metabolic pathways that function in different parts of the cell, including glycolysis, FA synthesis, and lipid synthesis. However, the precise molecular mechanism by which *BnLACS2* influences the expression of genes involved in those physiological processes should be further studied.

## Methods

### Plants materials and growth condition

Plants of *B. napus* (Zhongshuang 9) were obtained from the Oil Crops Research Institute (OCRI) of the Chinese Academy of Agricultural Sciences (CAAS) and were grown in the experimental fields of Jiangsu University, Zhenjiang, China. For tissue-specific expression analyses, rapeseed roots, stems, leaves, flowers and developing seeds were collected and stored at − 70 °C. Low (EM91) and high (EM102) oil-content canola lines were obtained from State Key Laboratory of Crop Genetics and Germplasm Enhancement, Nanjing Agricultural University, China. The seed tissues were harvested at 25, 35, 45, and 50 DAP. *Nicotiana benthamiana* stored in our laboratory were grown in a plant growth room for 5–6 weeks under a temperature-adjusted daytime/night cycle of 22/20 °C, and an illumination time of 16 h/d.

### Sequence analysis of *BnLACS2*

The full length coding region of *BnLACS2* was obtained using the protein sequence of *AtLACS2* (NP_175368) to search *Brassica* genome sequence databases (http://www.genoscope.cns.fr/blat-server/cgi-bin/colza/webBlat). Protein properties, including molecular weight, isoelectric point, sequence length, and amino acid composition were determined using the ProtParam tools in the ExPASy database (http://web.expasy.org/protparam/). Domain analysis was performed using the CDD database in the National Center for Biotechnology Information (NCBI, http://www.ncbi.nlm.nih.gov) and the ScanProsite Results Viewer in ExPASy (http://us.expasy.org/prosite/). The amino acid sequences of plant LACS were retrieved from nonredundant protein sequence databases using the blast method in NCBI and *Brassica* genome sequence databases. Multiple alignment of the amino acid sequences of LACS homologs were analyzed using the ClustalW program [53]. Phylogenetic analyses were performed using the Neighbor-joining algorithm in MEGA 5.0 software [54].

### Subcellular localization of *Bn*LACS2

Total RNA was extracted from the developing seeds of Zhongshuang 9 using the Trizol RNA Preparation Kit (Invitrogen, USA), following the manufacturer’s protocol. First-strand cDNA was synthesized using 1–2 μg of total RNA as the template, oligo (dT) as the primers, and reverse transcriptase (Fermentas). The cDNA of the *BnLACS2* gene was amplified by PCR using the primers, 5′-ATGTACACAAGCGGGACGAC-3′ and 5′-ACGCCAGTATCCAACAGAGG-3′, and cloned into the pMD18 T plasmid. To observe the subcellular localization of *Bn*LACS2, translational fusions of *BnLACS2*-pK7FWG2.0 and *CRT3*-pCX-DR were constructed and transformed into *Agrobacterium tumefaciens* strain GV3101 using the freeze-thaw method described in Karimi et al. [55]. The subcellular distribution of the transiently expressed *35S:BnLACS2-GFP* and *35S:DsRed-CRT3* in *N. benthamiana* was observed using a Leica TCS scanning confocal microscope, as described previously [56, 57].

### Yeast transformation and complementation

The cDNA of the *BnLACS2* gene was amplified using the primers, F: 5′-ggtaccATGGCTGCAGCTGCTGATCATG-3′ and R: 5′-ggatccCTAGCTCTGGACGCCTTTGCTTC-3′, and inserted into the multiple cloning sites (*Kpn I/BamH I*) of pYES2 to generate the pYES2-*BnLACS2* vector. The recombinant plasmid, pYES2-*BnLACS2*, and the pYES2 empty vector were introduced into the YB525 yeast strain by uracil auxotrophy [13, 58]. Then, positive recombinant clones were screened on Synthetic Dropout Medium-Uracil (SC/ura^−^) plates supplemented with 0.67% (w/v) Yeast nitrogen base lacking amino acids, with 0.077% (w/v) uracil default mixture, and 2% (w/v) glucose. Single colonies were randomly chosen and cultured in SC/ura- liquid medium to the logarithmic growth phase. Cells were then collected and incubated in 3–5 mL SC/ura^−^ liquid medium with 2% galactose for 4 h. After adjusting the cell concentration to a uniformity, 30 μL cultures were pipetted onto 3 ml SC/ura^−^ liquid medium containing 2% galactose plus 98 μM of different FAs, including 12:0 lauric acid, 14:0 myristic acid, 16:0 palmitic acid, 18:0 stearic acid, 18:1 oleic acid and 22:1 erucic acid dissolved in 0.1% Triton X-100 as the sole carbon source. Cultures were then cultivated for 84 h at 30 °C. The growth of yeast cells was estimated based on absorbance at 600 nm using a spectrophotometer.

### Analysis of lipids and FAs in yeast *pep4*

The recombinant vector, pYES2-*BnLACS2,* and the pYES2 control were transformed into *pep4*, a proteinase-deficient mutant strain. Screening of positive yeast monoclonal and induction of the *Bn*LACS2 protein are consistent with the methods described above. Yeast cell concentrations were adjusted to the same concentration based on the optical density at 600 nm. As described by Thakur et al. [59], equivalent volumes of yeast suspensions were then centrifuged to collect the cells, and stained with 0.3% Sudan black B to detect the neutral lipid content. The lipids were extracted from yeast cells using chloroform and methanol method as described by Bligh and Dyer [60]. Two dimensional thin layer chromatography (2D-TLC) was performed on 20 × 20 cm silica gel 60F254 aluminum sheets (Merck, Germany). The solvents were chloroform/methanol/water (65:30:2.5, v/v) in the first dimension and chloroform/methanol/acetic acid/water (80:12:15:4, v/v) in the second dimension. The polar lipids were detected by staining with chromogenic reagents as described by Guan et al. [61].

FAs were methylated following the procedure of Brandenburg et al. [62]. Prior to GC-MS analysis, 10 μl methylheptadecanoate (10 μg/μl, Sigma-Aldrich, USA) was added into the extracted lipid samples as an internal standard. FA profiles were determined using GC-MS equipped with a 30 m × 0.25 mm BPX 70 fused-silica capillary column (SGE, Austin, TX, USA), as previously described [63]. After the identification of each FA species based on their specific retention times, the FAs were quantified using the internal standard.

### Proteomics analysis of the *pep4* yeast strain transformed with the *BnLACS2* gene

After induction of the pYES2-*BnLACS2* and pYES2 vectors, total proteins were extracted from the *pep4* yeast strain using a Tris buffer-based protocol, as described by Isola et al. [64]. Protein concentration was determined by the Bradford method [65]. 2-DE was performed as described in Ding et al. [66], with a few minor changes. Protein samples were isolated in 17 cm pH 3–10 linear gel strip (Bio-Rad). Isoelectric focusing procedure was set at 250, 500 and 2000 V for 1 h, respectively, and then 8000 V for 56,000 Vh. After equilibration and the second dimension sodium dodecyl sulfate polyacrylamide gel electrophoresis was performed, gels were visualized using Coomassie Brilliant Blue R-250 (0.1%) staining and scanned using a SanMaker 9700XL instrument (Bio-Rad). Image analysis using PDQuest software (Bio-Rad) and in-gel digestion were performed as described in Ding et al. [67].

Spots with statistically significant changes (Student’s *t*-test, *P* < 0.05) above a 2-fold threshold were selected for mass spectrometric (MS) analysis using the UltrafleXtreme MALDI TOF/TOF instrument (Bruker Daltonics, Billerica, MA, USA). Mass data were acquired in the positive ion reflector mode. To identify differentially expressed proteins, the peptide mass fingerprint was searched in the NCBI non redundant (nr) database using Mascot software (http://www.matrixscience.com). The following search parameters were used: taxonomy, fungi; trypsin; one missed cleavage allowed; fixed modification, carbamidoethyl; variable modifications, dioxidation; peptide tolerance, ± 0.2 Da; and peptide charge, 1+. Protein scores > 65 were considered significant (*P* < 0.05).

### Generation of transgenic rapeseed plants

To generate CaMV 35S-driven constructs, the full-length coding region of *BnLACS2* was amplified using primers 5′-ggtaccATGGCTGCACTGCTGATCATG-3′ and 5′-ggatccCTAGCTCTGGACGCCTTTGCTTC-3′. The resulting fragment was digested with *BamH I* and *Kpn I*, and then inserted into the same sites of the pCAMBIA1300-35S-Nos vector. To construct the *BnLACS2*-RNAi plasmid, partial fragments of *BnLACS2* were amplified using two pairs of primers: 5′-ctcgagCAGCTATTTAGTGAGGCTGTGAA-3′, 5′-ggtaccGGAAGTGTCTTATCCGAGT-3′ and 5′-tctagaCAGCTATTTAGTGAGGCTGTGAA-3′, 5′-ggatccGGAAGTGCTGTCTTATCCGAGT-3′. The resulting fragments were digested with *Xho I*, *Kpn I* and *Xba I*, *BamH I*, respectively, and inserted into the same sites of the pKANNIBAL vector. The resulting recombinant plasmid was then digested with *Not I* and the fragment was inserted into pART27. All plasmids were verified by restriction digestion and DNA sequencing analysis.

The *BnLACS2*-OE and *BnLACS2*-RNAi constructs were introduced into the *A. tumefaciens* strain, GV3101, which were then used for the transformation. The transformation of canola plants was performed using the floral dip method [68]. Seeds were harvested from the transformed plant and screened for putative transformants. Positive *BnLACS2*-OE or -RNAi transgenic plants were first screened on selection medium containing hygromycin (100 μg/mL) or kanamycin (50 μg/mL), respectively, and then by PCR and RT-PCR using the primers presented in Additional file 5: Table S2).

### Determination of oil content and FAs in transgenic rapeseed

Seed oil content was measured using near infrared spectrometry (NIRS) and nuclear magnetic resonance (NMR), as described in Bellincontro et al. [69]. The seeds (about 2.0 g) of Zhongshuang 9 and independent T_2_ transgenic lines at the same growth stage were collected, and the oil contents were measured in triplicate. FA level was determined by GC-MS, as described above.

### Quantitative real-time PCR analysis

The qRT-PCR of first-strand cDNA was performed using SYBR® PremixExTaq™II (TaKaRa, Japan), according to Wang et al. [70]. After optimizing the qRT-PCR conditions, the amplification efficiency was above 90% based on standard curves, Ct values ranged from 20.9 to 29.3, and all melt curves displayed a single specific peak. The relative expression of the target genes was normalized to the expression data of *BnACTIN2* using the 2^-∆∆CT^ method [71]. All qRT-PCRs were performed in triplicate. The primers used for qRT-PCR analyses are listed in Additional file 6: Table S3.

## Supplementary information

**Additional file 1: Figure S1.** Full length cDNA and deduced amino acid sequences of *BnLACS2*. Exons are indicated by black and red lines.

**Additional file 2: Figure S2.** Expression of *BnLACS2* increased the lipid (a) and FAs (b) contents in yeast**.** Neutral lipids of pYES2 and pYES2-*BnLACS2* transformants are stained with Sudan Black B and the absorbance is measured at 580 nm, respectively (left). Polar lipids are detected by 2D-TLC (right). The circle indicates the phospholipids induced in pYES2-*BnLACS2* transformant. The Error bars indicate SD (*n* = 3). The significant differences between pYES2 and pYES2-*BnLACS2* transformants are indicated (Student’s *t*-test): ***, *P* < 0.001; **, *P* < 0.01; *, *P* < 0.05.

**Additional file 3: Figure S3.** 2-DE maps of total proteins in pYES2-*BnLACS2* (a) and pYES2 transformants (b). The representative images from three biological replicates are shown. The arrows in images indicate 13 differentially expressed proteins that changed reproducibly and significantly in pYES2-*BnLACS2* compared with pYES2.

**Additional file 4: Table S1.** Protein identities of the differentially expressed protein spots.

**Additional file 5: Table S2.** Primer sequences used for transgenic plants detection.

**Additional file 6: Table S3.** Primer sequences used for quantitative real-time PCR.

## Data Availability

The datasets used and/or analyzed during the current study are available from corresponding authors on reasonable request.

## Abbreviations

*ACC1*: *acetyl-CoA carboxylase 1*
CRT: Calreticulin
*DGAT1*: *diacylglycerol acyltransferase 1*
*ENO*: *enolase*
ER: Endoplasmic reticulum
*FAD3*: *FATTY ACID DESATURASE 3*
*FAE1*: *FATTY ACID ELONGATION1*
FAs: Fatty acids
G3P: glycerol-3-phosphate
*KASII*: *beta-ketoacyl-[acyl carrier protein] (ACP) synthase*
LACSs: Long-chain acyl-coenzyme A synthetases
*LPAT1*: *lysophosphatidyl acyltransferase 1*
*PFK*: *phosphofructo kinase*
*PGK*: *phosphoglycerate kinase*
*PYK*: *pyruvate kinase*
TAGs: Triacylglycerols
