# Supplementary material for: Long-chain acyl-CoA synthetase 2 is involved in seed oil production in Brassica napus
Source: BMC Plant Biol. 2020 Jan 13;20:21. doi: 10.1186/s12870-020-2240-x (PMC6958636; doi:10.1186/s12870-020-2240-x)
Supplement: Supplementary file 1 — Additional file 1: Figure S1. Full length cDNA and deduced amino acid sequences of BnLACS2. Exons are indicated by black and red lines. [file 12870_2020_2240_MOESM1_ESM.docx]

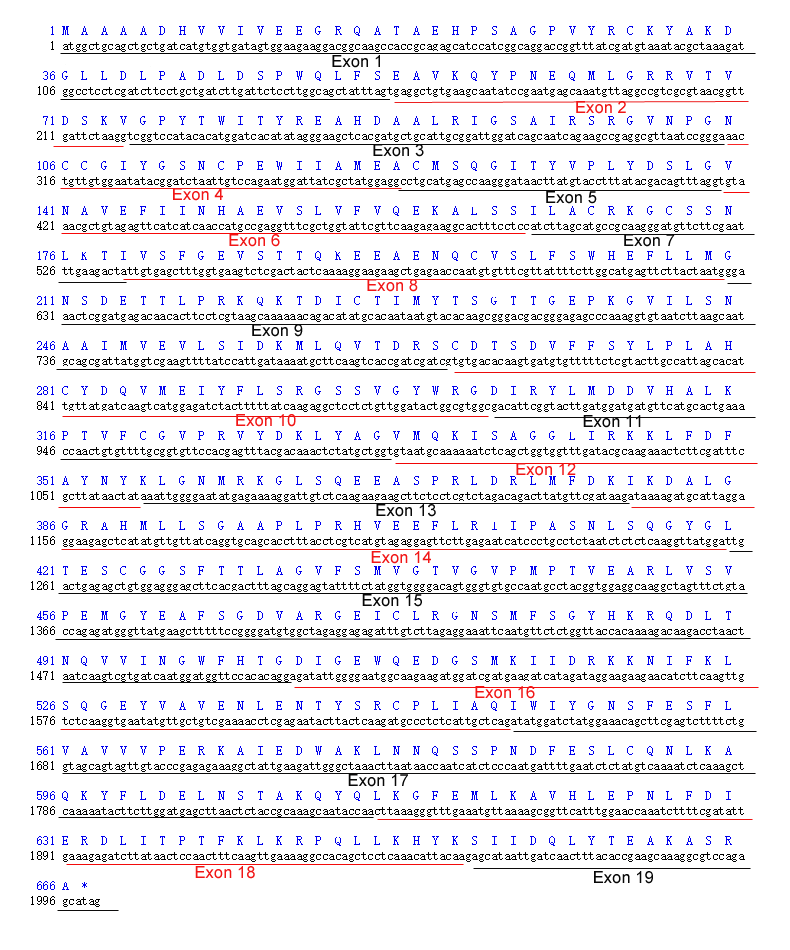


**Figure S1.** Full length cDNA and deduced amino acid sequences of *BnLACS2*. Exons are indicated by black and red lines.
