# Supplementary material for: Long-chain acyl-CoA synthetase 2 is involved in seed oil production in Brassica napus
Source: BMC Plant Biol. 2020 Jan 13;20:21. doi: 10.1186/s12870-020-2240-x (PMC6958636; doi:10.1186/s12870-020-2240-x)

**Figure S2.** Expression of *BnLACS2* increased the lipid (a) and FAs (b) contents in yeast**.** Neutral lipids of pYES2 and pYES2-*BnLACS2* transformants are stained with Sudan Black B and the absorbance is measured at 580 nm, respectively (left). Polar lipids are detected by 2D-TLC (right). The circle indicates the phospholipids induced in pYES2-*BnLACS2* transformant. The Error bars indicate SD (n=3). The significant differences between pYES2 and pYES2-*BnLACS2* transformants are indicated (Student’s *t*-test): ***, *P* < 0.001; **, *P* < 0.01; *, *P* < 0.05.


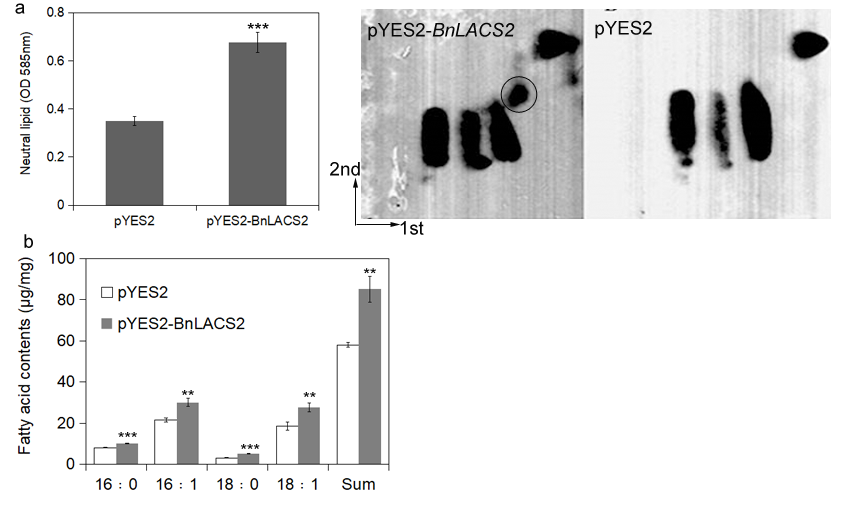

Supplement: Supplementary file 2 — Additional file 2: Figure S2. Expression of BnLACS2 increased the lipid (a) and FAs (b) contents in yeast. Neutral lipids of pYES2 and pYES2-BnLACS2 transformants are stained with Sudan Black B and the absorbance is measured at 580 nm, respectively (left). Polar lipids are detected by 2D-TLC (right). The circle indicates the phospholipids induced in pYES2-BnLACS2 transformant. The Error bars indicate SD (n = 3). The significant differences between pYES2 and pYES2-BnLACS2 transformants are indicated (Student’s t-test): ***, P < 0.001; **, P < 0.01; *, P < 0.05. [file 12870_2020_2240_MOESM2_ESM.docx]
