# Supplementary material for: Long-chain acyl-CoA synthetase 2 is involved in seed oil production in Brassica napus
Source: BMC Plant Biol. 2020 Jan 13;20:21. doi: 10.1186/s12870-020-2240-x (PMC6958636; doi:10.1186/s12870-020-2240-x)
Supplement: Supplementary file 3 — Additional file 3: Figure S3. 2-DE maps of total proteins in pYES2-BnLACS2 (a) and pYES2 transformants (b). The representative images from three biological replicates are shown. The arrows in images indicate 13 differentially expressed proteins that changed reproducibly and significantly in pYES2-BnLACS2 compared with pYES2. [file 12870_2020_2240_MOESM3_ESM.docx]

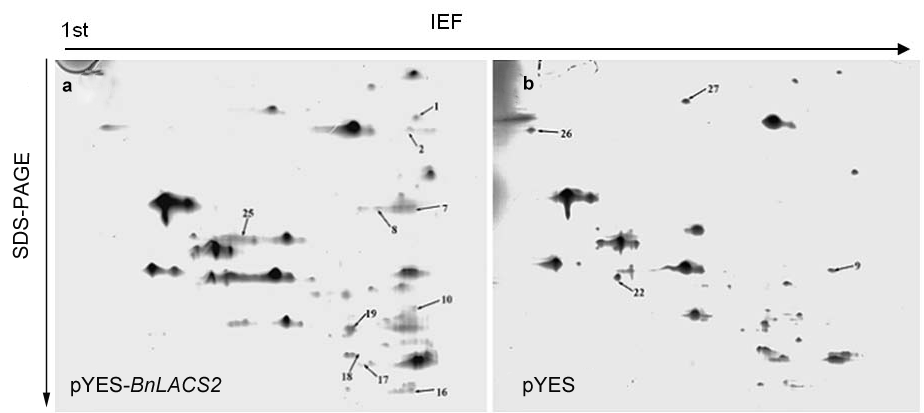
**Figure S3.** 2-DE maps of total proteins in pYES2-*BnLACS2* transformants (a) and pYES2 (b). The representative images from three biological replicates are shown. The arrows in images indicate 13 differentially expressed proteins that changed reproducibly and significantly in pYES2-*BnLACS2* compared with pYES2.
