# Supplementary material for: Long-chain acyl-CoA synthetase 2 is involved in seed oil production in Brassica napus
Source: BMC Plant Biol. 2020 Jan 13;20:21. doi: 10.1186/s12870-020-2240-x (PMC6958636; doi:10.1186/s12870-020-2240-x)
Supplement: Supplementary file 4 — Additional file 4: Table S1. Protein identities of the differentially expressed protein spots. [file 12870_2020_2240_MOESM4_ESM.docx]

**Table S1.** Protein identities of the differentially expressed protein spots.

| **Spot No.** | **MW/pI** | **NCBI accession** | **Protein name** | **Score** | **Species** |
| --- | --- | --- | --- | --- | --- |
|  |  |  |  |  |  |
| **Up-regulated proteins** | | | | | |
| 1 | 61.3/5.5 | gi\|50516875 | Hypothetical protein | 67 | *Debaryomyces hansenii* CBS767 |
| 2 | 26.7/8.3 | gi\|15788446 | Phosphoglycerate mutase | 134 | *Saccharomyces cerevisiae* |
| 7 | 35.8/6.5 | gi\|6321631 | Glyceraldehyde 3-phosphate dehydrogenase | 96 | *Saccharomyces cerevisiae* |
| 8 | 46.7/6.2 | gi\|20151217 | Yeast enolase1 | 104 | *Saccharomyces cerevisiae* |
| 10 | 61.7/5.8 | gi\|7245976 | Pyruvate decarboxylase | 67 | *Saccharomyces cerevisiae* |
| 16 | 65.1/5.2 | gi\|29420851 | Vacuolar membrane ATPase | 69 | *Saccharomyces cerevisiae* |
| 17 | 66.7/5.3 | gi\|6319972 | Cytoplasmic ATPase | 143 | *Saccharomyces cerevisiae* |
| 18 | 55.0/8.0 | gi\|4180 | Pyruvate kinase | 164 | *Saccharomyces cerevisiae* |
| 19 | 46.9/5.7 | gi\|6321968 | Eno2p | 148 | *Saccharomyces cerevisiae* |
| **Down-regulated proteins** | | | | | |
| 9 | 55.0/6.7 | gi\|119389904 | Mitochondrial F1-ATPase | 96 | *Saccharomyces cerevisiae* |
| 22 | 37.2/6.2 | gi\|112491285 | Alcohol dehydrogenase 1 | 194 | *Saccharomyces cerevisiae* |
| 26 | 26.8/5.8 | gi\|230405 | Triosephosphate isomerase | 197 | *Saccharomyces cerevisiae* |
| 27 | 24.0/5.3 | gi\|295614 | Heat shock protein 26 | 214 | *Saccharomyces cerevisiae* |
