# Supplementary material for: Long-chain acyl-CoA synthetase 2 is involved in seed oil production in Brassica napus
Source: BMC Plant Biol. 2020 Jan 13;20:21. doi: 10.1186/s12870-020-2240-x (PMC6958636; doi:10.1186/s12870-020-2240-x)
Supplement: Supplementary file 5 — Additional file 5: Table S2. Primer sequences used for transgenic plants detection. [file 12870_2020_2240_MOESM5_ESM.docx]

**Table S2** Primer sequences used for transgenic plants detection

| **Name** | **Forward primer** | **Reverse primer** |
| --- | --- | --- |
| Overexpreesion | 5'-AGGGTCTTGCGAAGGATAG-3' | 5'-ATGTACACAAGCGGGACGAC-3' |
| *RNAi* | 5'-TACCCGAGTAACAATCTCCAGG-3' | 5'-ggtaccGGAAGTGCTGTCTTATCCGAGT-3' |
| *RT-RCR* | 5'-ATGTACACAAGCGGGACGAC-3' | 5'-ACGCCAGTATCCAACAGAGG-3' |
| *BnACTIN* | 5'-GTTGCTATCCAGGCTGTTCT-3' | 5'-ACTGCTCTTAGCCGTCTCC-3' |
