# Supplementary material for: Long-chain acyl-CoA synthetase 2 is involved in seed oil production in Brassica napus
Source: BMC Plant Biol. 2020 Jan 13;20:21. doi: 10.1186/s12870-020-2240-x (PMC6958636; doi:10.1186/s12870-020-2240-x)
Supplement: Supplementary file 6 — Additional file 6: Table S3. Primer sequences used for quantitative real-time PCR. [file 12870_2020_2240_MOESM6_ESM.docx]

**Table S3** Primer sequences used for quantitative real-time PCR

| **Name** | **Forward primer** | **Reverse primer** |
| --- | --- | --- |
| *BnLACS2* | 5'-ATGTACACAAGCGGGACGAC-3' | 5'-ACGCCAGTATCCAACAGAGG-3' |
| *PFK* | 5'-ATTAGGGAAATCGTGAGCAG-3' | 5'-CCGTTCTCTATACTCTCGGC-3' |
| *PGK* | 5'-AGGCACAAGGTCTGTCTGTTGGAT-3' | 5'-AGCGAACTTGTCAGCAACAACAAC-3' |
| *ENO* | 5'-CTGATTATCTC GGAAAGGGTG-3' | 5'-GAGGAATGCCACTGACAACG-3' |
| *PYK* | 5'-AAAGGCTGGTTTGAACATTG-3' | 5'-ATTTCGTGGTTTGGTGGG-3' |
| *ACC1* | 5'-AATATAACAGATGCGCCTCG-3' | 5'-TGCAAGACAATCCAGCTTAC-3' |
| *KASⅡ* | 5'-GCTAATACCACTGTTGCCTT-3' | 5'-CTCTCTCTCTCTCAGCACTC-3' |
| *FAE1* | 5'-TCTCCGCGATGGTCGTTAACACTT-3' | 5'-TCCTTGGACAAACTCACTCCGGTT-3' |
| *FAD3* | 5'-TATAAGGGCGGCCATTCCTAAGCA-3' | 5'-AGATAGCCCAGAACAGGGTTCCTT-3' |
| *LPAT1* | 5'-CGCCATTGTTCTCATCGTC-3' | 5'-GCCAAGGCTGAGAAGTGTG-3' |
| *DGAT1* | 5'-TTCACTTTCGGTCTTTCCTTT-3' | 5'-CCAGGGTTCTTATGTCGTAGC-3’ |
| *BnACTIN* | 5'-GTTGCTATCCAGGCTGTTCT-3' | 5'-ACTGGTCTCTTAGCCGTCTCC-3' |
